# Allometric and Non-Allometric Patterns in Sexual Dimorphism Discrimination of Wing Shape in *Ophion intricatus*: Might Two Male Morphotypes Coexist?

**DOI:** 10.1673/031.013.14301

**Published:** 2013-12-04

**Authors:** Hugo A. Benítez, Raffaella Bravi, Luis E. Parra, Maria-Jose Sanzana, Einer Sepúlveda-Zúñiga

**Affiliations:** 1 Faculty of Life Sciences, University of Manchester, Michael Smith Building, Oxford Road, Manchester M13 9PT, UK; 2 Instituto de Alta Investigación, Universidad de Tarapacá, Casilla 7-D Arica, Chile; 3 Environmental Biology Department, University Roma Tre, V.le G. Marconi 446, 00146, Rome, Italy; 4 Departamento de Zoología, Facultad de Ciencias Naturales y Oceanográficas, Universidad de Concepción, Concepción, Casilla: 160-C - 4070386, Chile

**Keywords:** allometry, geometric morphometrics, sexual selection, sexual shape dimorphism, wasp

## Abstract

Bees and wasps could exhibit shape and size sexual dimorphism, and most of their morphological variation could depend on phenotypic responses due to environmental pressure during ontogenetic development. More complex measurement techniques related to size and shape rather than simply to mass and length should be required to analyze such a complex sexual dimorphism. In this study, differences related to wing shape and size of males and females of *Ophion intricatus* Brullé (Hymenoptera: Ichneumonidae) were evaluated using geometric morphometrics. Right and left wings of specimens were used, and a photographic matrix was constructed in which 18 morphological landmarks based on shape and vein patterns of the wings were digitalized. A multivariate analysis of wing shape showed significant differences between sexes and sites. The geometric variation demonstrated that the points at the intersection of radial and cubital-anal veins might be key characters to differentiate between sexes. This study also showed the presence of two clearly different male morphotypes coexisting in the same study site. However, it should be noted that the results of this study showed that the variation in wing shape is an analytical character in the determination of sexual differences in the family Ichneumonidae. These differences raise the question of whether sexual dimorphism of wing shape may be modulated by natural selection.

## Introduction

Bees and wasps, as most insects, exhibit sexual size and shape dimorphism (e.g., Hurlbutt 1987; Mackauer 1996; O'Neill 2001; Pretorius 2005; Mitrovski-Bogdanović et al. 2009; Zikic et al. 2009). Most of the morphological variation in wasps could be due to phenotypic responses (plasticity) that act during ontogenetic development as a consequence of environmental pressures (Shreeves and Field 2008). Females have some adaptive advantages over males, such as greater fecundity and parental care, because females are typically larger than males (Forrest 1987; Andersson 1994; Moller and Zamora-Muñoz 1997; Reeve and Farbain 1999). However, in some species, males are longer but have less mass than females (e.g., Cepeda-Pizarro et al. 1996). Those considerations suggest that inferring and quantifying sexual dimorphism requires more complex measurement techniques related to shape and size rather than simply to mass and length (Gidaszewski et al. 2009; Benítez et al. 2010). Another important factor to be evaluated within sexual dimorphism is allometry, defined as the association between size and shape, or the covariation of parts due to variation in size. All types of allometry can contribute to the sexual differentiation at the respective levels of variation (Gidaszewski et al. 2009). Allometry is classified according to the cause of variation in size that gives rise to the allometric relationship, which is usually ontogenetic growth or evolutionary change; therefore, there is either ontogenetic or evolutionary allometry, respectively. If individuals of the same species are examined at the same ontogenetic stage, both of these sources of “dynamic” variation are kept constant, and the remaining individual variation would be “static,” corresponding to static allometry (Klingenberg 1996).

Ophionines are solitary koinobiont endoparasitoids of the larvae of other holometabolous insects. Many ophionines oviposit into relatively mature larvae in their 3rd or 4th (Vickery 1929) or 4th or 5th instar (Moutia and Courtois 1952; Yu and Horstmann 1997), though some species attack young larvae (Price 1975). Generally a single egg is deposited free in the haemocoel. No species of ophionine are known to develop gregariously (Gauld and Bolton 1988; Yu and Horstmann 1997). The genus *Ophion* Fabricius (Hymenoptera: Ichneumonidae) includes about 200 species (Gauld 1978) distributed worldwide, and most of them inhabit temperate climates (Townes 1971). Most *Ophion* species are parasitoids, laying their eggs in Lasiocampidae, Noctuidae, and Notodontidae pupae or larvae.

In the present study, static allometry is analysed on the species *Ophion intricatus* Brullé (Hymenoptera: Ichneumonidae). Geometric morphometrics techniques were used, with the aim to investigate sexual dimorphism of wings and to characterize the wings as a trait of sexual differentiation, as well as to show that there is a differentiation pattern in males of the same species coexisting in the same habitat. This is the first step for further studies on behavior at group level in the area.

## Materials and Methods

### Data acquisition

A total of 136 specimens of *O. intricatus* (50 female and 86 males) were used in this study, and they were collected in the Botanical Park “Pedro del Rio Zañartu” - Bio Bio Region, Chile (36° 47′ S, 73° 09′ W). Wasps were collected with UV light traps (GE Power Pro 950, www.ge.com) using an 800- watt electric generator. The light sources were placed over a white sheet to increase the luminosity. Traps were installed for 4 hr in different sampling points. Following the key of Gauld (1978), specimens were carefully checked for their species status, and the process was based on taxo-morphological characters. Once collected, specimens were processed and mounted. Right and left wings were removed from the body and then mounted in fixed molds (compressed between two microscope slides) for subsequent image capturing.

### Morphometric analysis

**Figure 1.**
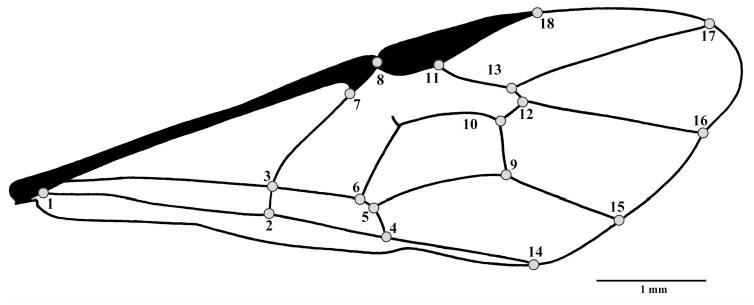
Representation of 18 morphological landmarks in the wing of *Ophion intricatus.* High quality figures are available online.

Right and left wings of wasps were photographed with a Sony 10 DSC-H7 camera (www.sony.com) with directed fiber optics lighting. Then, photographic matrixes were constructed using TpsUtil 1.57 program (Rohlf 2008). Eighteen morphological landmarks (Type 1 landmarks) were digitized for left and right wings in all specimens, selected according to external anatomy and vein pattern of the wings (Figure 1, Table 1) (Zelditch et al. 2012). For this purpose, TpsDig 2.12 software (Rohlf 2008b) was used. The x-y coordinates of the biologically homologous landmarks were then aligned and superimposed with the method of minimum least squares based on the generalized procrustes analysis (Dryden and Mardia 1998; Rohlf and Slice 1990).

Once the Cartesian x-y coordinates for all points were obtained, shape information was extracted from the coordinate data by using a full Procrustes fit (Rohlf and Slice 1990; Dryden and Mardia 1998). Due to the matching symmetry of the wings, the dataset contained two separate landmark configurations, one for the left and one for the right side. The analysis of shape included the reflection of all configurations from one body side to its mirror image (for details see Klingenberg and McIntyre 1998; Klingenberg et al. 2002). After reflection, all configurations can be superimposed in the Procrustes fit, a procedure that removes in formation on location orientation and rotation and standardizes each specimen to unit centroid size, which is the square root of the summed squared Euclidean distances from each landmark to the specimen centroid and provides an estimate of the size of the studied structure (Dryden and Mardia 1998). Centroid size was used as a measure of whole body size, while the information about the shape variation was extracted from the Procrustes superimposition.

**Table 1.**
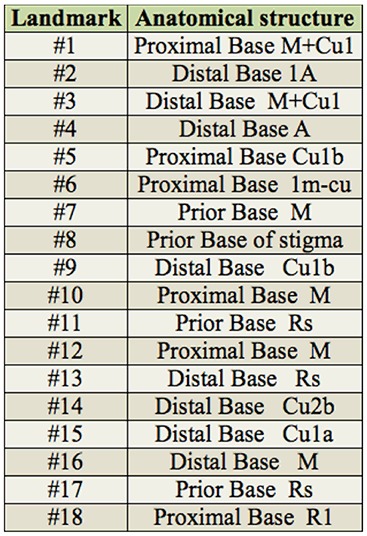
Anatomical description of landmarks in the wing of *Ophion intricatus*. *A* = Anal; *Cu* = Cubital; *M* = Medium; *R* = Radial; *Rs* = Sector radial; *M+Cu* = Medium cubital; *m* = Medium transversal; *cu* = Cubital transversal; *m+cu* = Medium cubital transversal.

After performing the Procrustes fit, the covariance matrix generated from the Procrustes coordinates, and which included the measures of the association between Procrustes coordinates (the x-y coordinates of each landmark after the Procrustes superimposition), were extracted. Shape variation in the entire dataset was then analyzed with principal component analysis (PCA) based on decomposition of covariance matrix.

Additionally, to assess shape and size variation between sexes, Procrustes ANOVA was performed, and the results are reported as sums of squares and mean squares that are dimensionless (Klingenberg and McIntyre 1998; Klingenberg et al. 2002).

Static allometry was evaluated by a multivariate regression of shape, pooled within sex (Procrustes coordinates), on centroid size. Then, the predicted values were used as shape variables, accounting for the allometric component of shape variation, and the residuals were used as the non-allometric component of shape variation. A plot of the regression scores (the projection of shape data onto the direction of the regression vector in the shape tangent space) was performed to better visualize allometric relarelationships (Drake and Klingenberg 2008; Klingenberg et al. 2012). All morphometric and statistical analyses were performed using the free morphometric software Morpho J 1.05f (Klingenberg 2011).

**Table 2.**
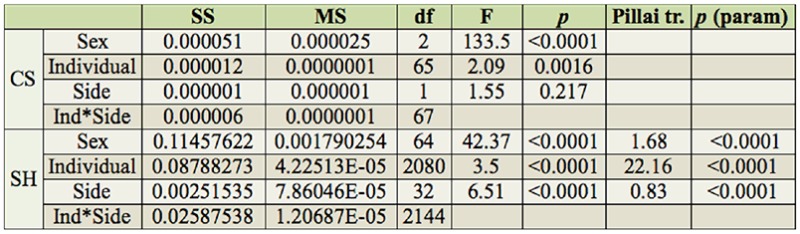
Procrustes ANOVA for both centroid size (CS) and shape (SH) of *Ophion intricatus*. Sums of squares (SS) and mean squares (MS) are in units of Procrustes distances (dimensionless).

## Results

**Figure 2.**
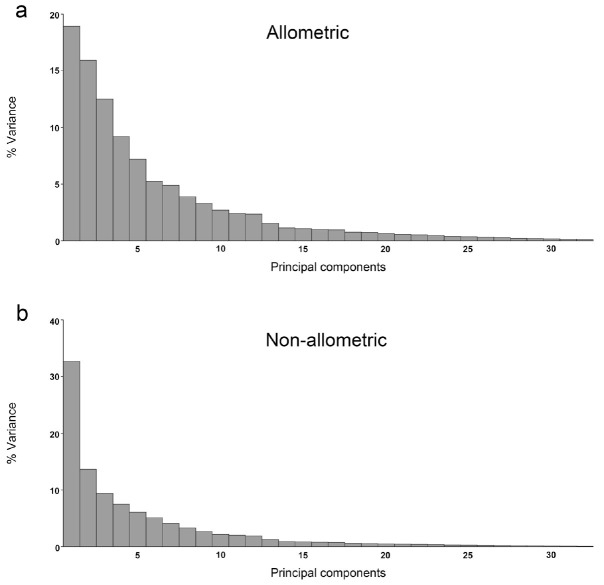
Total shape variation by PCA is shown, using covariance matrices of a) individual variation (allometric component) and b) corrected by size (non-allometric component). High quality figures are available online.

PCA for allometric and non-allometric components of within-sex variation, showed how the first principal components accumulated shape variation in very few dimensions (Figure 2).

Regarding allometric components, the first three PCs accounted for 46.6% (PC1 + PC2 + PC3 = 18.178% + 16.365% + 12.061%) of the total shape variation, and provided a reasonable estimation of the total variation. The other PCs each accounted for no more than 8.8% of the variation.

**Figure 3.**
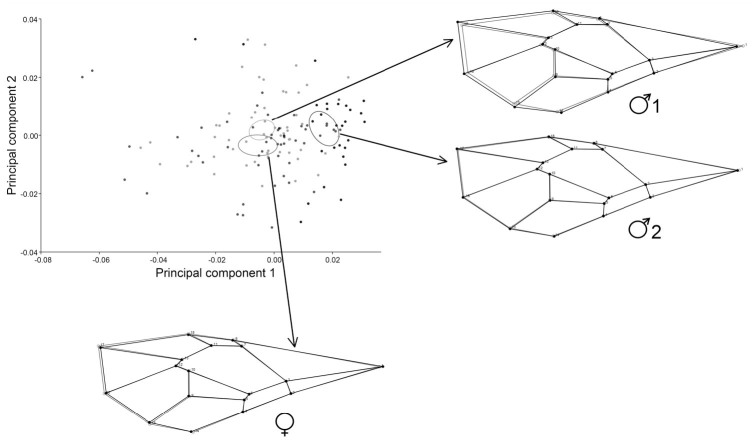
PCA of wing shape variation (allometric component, pooled within sexes). The different confident ellipses show females and two male morphotypes with a 95% level of confidence. In the x and y axes, PC1 and PC2 are respectively shown with associated shape deformation images. High quality figures are available online.

**Figure 4.**
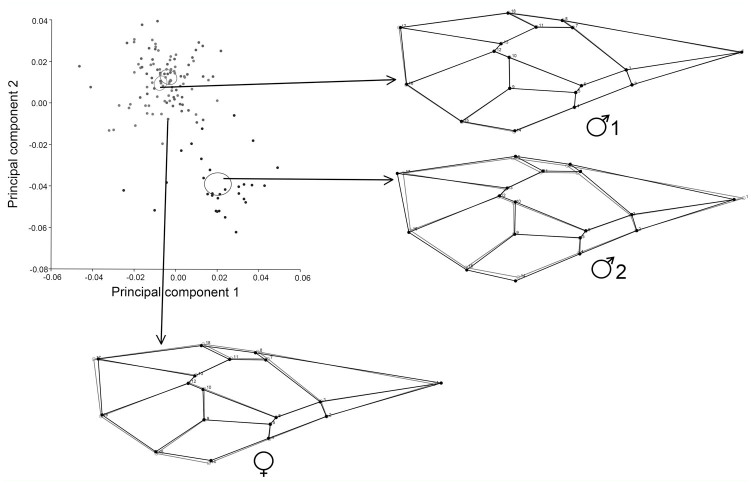
PCA of wing shape variation (non-allometric component, pooled within sexes). The different confident ellipses show the female and two male morphotypes with a 95% level of confidence. The first two PC axes with shape deformation images associated are shown. High quality figures are available online.

Regarding non-allometric components, PC1 held more than 30% of the complete variation between sexes, and the remnant PCs each accounted for no more than 13.6% (Figure 3, 4). Procrustes ANOVA showed that variation in size and symmetric shape between sexes was highly significant (Table 2). To quantify static allometry, multivariate regression of shape (pooled within sexes) was used on centroid size. The results confirmed the occurrence of a strong static allometric effect that accounted for 30.48% of the total shape variance (*p* < 0.0001).

## Discussion

The results of this study confirmed the presence of sexual variation in wing shape in *O. intricatus*. A number of studies have found that sexual dimorphism associated with size and shape in insects is adaptive (Nylin and Gotthard 1998; Farbain et al. 2007; Gidaszewski et al. 2009; Benitez et al. 2013). Some of the hypotheses proposed to explain this kind of dimorphism in arthropods do not take into account whether the differences could be due exclusively to sexual dimorphism or to allometric processes (Walker and Rypstra 2001; Esperk et al. 2007; Gidaszewski et al. 2009). According to Zikic et al. (2010), allometry contributes to the variation in body shape among female biotypes of *Ephedrus persicae*. Several morphological characters, such as size of head, width of petiolus, width of mesoscutum, and length of ovipositor sheath, diverged among the *E. persicae* biotypes. In our study, the influence of size on sexual differences was taken into account, and also if there was some effect on the shape differentiation (Klingenberg 1996; Fairbairn 1997; Debat et al. 2003).

The wings of *O. intricatus* were evaluated, and it was determined that they were strongly under sexual dimorphism. The results show that females tended to have longer wings than males, as seen in the displacement of landmarks 4, 7, and 8 (the connection of the transversal radial and cubital- anal veins). This type of wing dimorphism is present in almost all parasitoids within Ichneumonidae, as the females' metasomas are likely to be a bit heavier than males because the females carry eggs. This extra weight could result in slight compensatory differences in wing size and shape. This kind of variation may be an adaptive response to flight, and for behavioral activities such as localization, foraging, and oviposition. In contrast, males' wings are wider, which might be linked to the selection of courting sites (longer flight periods in search of sexually active females) and to predator avoidance.

A differentiation of wing shape between two male populations in the same sampling site was found, and two male morphotypes seemed to coexist. The presence of these two male morphotypes might be the effect of sexual differentiation due to females not preferring a particular male wing shape. However, it must be taken into account that male populations used different microhabitats. The light traps used could reasonably have attracted specimens from different microhabitats during the collecting process, and males may have morphologic microadaptation to their habitat. Zikic et al. (2010) reported that wing shape characters related to allometry differed among female specimens of *E. persicae* that emerged from different aphid hosts, indicating differences in their ecology and physiology.

It is expected that the anatomical design of the wings of insects will be subjected to strong selection. Here, several wing morphotypes emerged, raising the question of whether sexual dimorphism on wing shape may be modulated by natural selection (Betts and Wootton 1988; Dudley 2000).

Behavioral studies are needed in order to clarify what the adaptive significance of the findings of our study are. However, it is worth noting that the results of this study showed that variation in wing shape was an analytical character in the determination of sexual differences in the individuals of *O. intricatus*.

## Abbreviations

PCA,: principal component analysis
